# How do government health departments in Australia access health economics advice to inform decisions for health? A survey

**DOI:** 10.1186/1743-8462-6-6

**Published:** 2009-04-09

**Authors:** Lynne Madden, Lesley King, Alan Shiell

**Affiliations:** 1 Public Health Medicine Training and Development, Australasian Faculty of Public Health Medicine, 145 Macquarie Street, Sydney, NSW, 2000, Australia; 2 Prevention Research Collaboration, School of Public Health, University of Sydney, Level 2, K25, Medical Foundation Building, 92-94 Parramatta Road, Sydney, NSW 2006, Australia; 3 Population Health Intervention Research Centre, University of Calgary, 3330 Hospital Drive NW, Calgary, T2N 4N1, Alberta, Canada

## Abstract

**Background:**

Government anticipates that health economic analysis will contribute to evidence-based policy development. Early examples in Australia where this expectation has been met include the economic evaluations of breast and cervical screening. However, the level of integration of health economics within health services that require this advice appears uneven. We sought to describe how government health departments in Australia use specialist health economic advice to inform policy and planning and the mechanisms through which they access this advice.

**Methods:**

Information describing the arrangements for gaining health economics input into health decision-making was sought through interviews with a purposeful sample of economists and non-economists employed by all departments of health in Australia (state, territories and national). The survey was undertaken in August 2004. To aid interpretation of the results eight health economic functions were identified. As a comparison, four other government departments in NSW provided information about their access to economic advice.

**Results:**

All health departments except one reported being current users of health economics expertise. A variety of arrangements were described to source this, from building organisational capacity with self-sufficient in-house units to forging links with external sources. However, specialist positions for economists or health economists employed within health were few. A framework mapping these arrangements for sourcing advice with the eight common health economic functions to be met is presented. All other non-health government departments approached accessed economic advice, with three having in-house units.

**Discussion:**

A small health economics capacity in Australia has been established over the past 30 years through a variety of structural and strategic mechanisms. Health departments value health economic advice and use a variety of arrangements to obtain this. These arrangements have strengths and weaknesses depending upon the task to be undertaken. The lack of uniformity of approach suggests that health departments are still seeking the best ways to incorporate this form of specialist advice into mainstream decision-making.

**Implications:**

Summarises ways that governments source specialist services. Demonstrates how to describe an organisation's need for specialist services as a set of functions. This approach could be applied to assessing need for other specialist areas of advice.

## Background

A broad range of information is required to analyse patterns of health and disease, inform health policy, and manage services and programs that treat and prevent ill health. Consequently, health and public health organisations draw expertise from many disciplines and professional groups [1]. One such discipline is health economics, which applies the principles of economics to decision-making in health care, and in so doing has challenged many of the conventional ways of thinking about health and the distribution of the finite public resources that fund public health services.

Health economics is a young field. In Australia, the first health economics research unit was established in 1978 at the Australian National University and was funded by the National Health and Medical Research Council (NHMRC) [2]. Following Kerr White's review of public health research and education in 1985, which encouraged the development of the capacity for "population-based' thinking, a sustained capacity in health economics began to be established in the 1990s [3,4]. The NHMRC and VicHealth funded the National Centre for Health Program Evaluation in Melbourne, and in Sydney, the Centre for Health Economics Research and Evaluation was supported by the NSW Department of Health, and from 1994, also by the Public Health Education and Research Program (PHERP) as a national specialist centre in health economics [5,6]. From these beginnings, today there are number of groups specialising in health economics around the country, usually located within universities.

Despite the promise held out by health economics as an aid to decision-making, policy-makers continue to experience problems accessing economic advice. This need was highlighted most notably in the United Kingdom (UK) by Derek Wanless in his report to HM Treasury [7]. Wanless considered that the information base for public health generally was poor; and while there was often evidence on the scientific need for action, there was little evidence describing the cost-effectiveness or the outcomes of the implementation of programs. He also noted a relatively slow acceptance of economic perspectives within public health.

In Australia, health economics has made a contribution to evidence-based policy development within public health. Early examples include the economic evaluations of breast and cervical screening that informed national screening policy [8,9]. Estimates of the social costs of smoking and the social benefits of reducing the number of people who smoke have provided support for effective anti-smoking policies and interventions in NSW [10,11] and nationally [12]. The evaluation of Australia's investment in the prevention and treatment of HIV/AIDS and Hepatitis C has also confirmed both the personal- and system-level savings resulting from these policies and underpinned support for continued action [13,14]. More recently, Australia has led international efforts to improve health systems resource allocation that pioneers the use of economic evaluation to guide decisions relating to new pharmaceuticals and new health technologies [15,16].

Nonetheless, despite these successful examples of utilising health economic expertise, there is the perception that health economic advice is not always well-matched to the needs of government policy-makers [17-19]. Further reviews of the public health workforce in Australia since 1990 continue to acknowledge health economics as a public health speciality skill that needs strengthening [20-22].

To explore this issue, we describe how departments of health around Australia, and some other government departments in New South Wales (NSW), access economic advice to inform policy and programs. To identify the potential strengths and weaknesses of these mechanisms they are mapped against the common functions that health economics expertise could be anticipated to fulfil.

## Methods

To identify the range of mechanisms that government health departments employ to access economic advice, we surveyed a non-random (purposively selected) sample of health economists and non-health economists employed by departments of health in all the states and territories of Australia and by the Australian Government Department of Health and Ageing. Initial contacts were identified through the networks of the authors and then by snowballing if necessary [23]. We interviewed people with knowledge of the health economics capacity within their jurisdiction.

Semi-structured interviews were conducted by telephone, where possible, or via e-mail where preferred by respondents. All interviews (*n *= 15) were conducted in August 2004 by one of the authors (LK). Information was sought about mechanisms for gaining health economics input into health decision-making in general, not just for public health issues.

The questions asked were:

1. What health economics capacity does your health department have in-house?

2. How do you gain health economics advice to inform decision-making?

3. What avenues are open to you to gain such advice?

In addition, to provide a point of comparison, information about arrangements used by four other government departments in NSW to obtain economic advice was obtained through an examination of websites or by telephone interview.

To describe common health economics functions we examined the Health Economics Competency Area developed for the competency framework that underpins delivery of the NSW Public Health Officer Training Program, and the self-assessment questions developed by the Canadian Health Services Research Foundation for use by decision-makers to assess their organisation's capacity to use research (or other specialised expertise) [24,25]. The organisational mechanisms identified by the survey were then mapped against these functions to determine the potential utility of the various mechanisms to obtain health economics advice.

## Results

The set of eight health economic functions identified are presented in Table 1. To make explicit what is meant by each function, illustrative questions or situations are provided in the table.

**Table 1:** Eight common health economics functions and examples of the types of situation in which they might be useful or types of questions that might be answered using this function

**Function**	**Types of questions/situation in which this** **function would be applied**
1. Appreciation of how economics fits into multi-disciplinary analysis of public health problems	Does this problem have an economic aspect? Would it benefit from an economic perspective?

2. Advanced appreciation of economic concepts and frameworks, able to frame issues, formulate questions and obtain advice	Problem has an economic aspect that can be framed i.e., the person is able to formulate an economic question in an appropriate way as part of a proposal.

3. Economic analysis of simple problems and issues, requiring literature searches, appraisal, synthesis and interpretation	Able to read and interpret the economic literature and think from an economic perspective.

4. A capacity to respond quickly to emerging and emergency issues	An economic surge capacity exists.

5. Conducting economic evaluations and other studies, with appropriate methods	Able to answer questions about performing economic analysis for example: when is the right time; how should it be done; what level of complexity is required; do we have the necessary skills and experience required, or do we know who has the necessary skills?

6. Application of economic findings to priority settings, emerging issues and decision-making	Able to apply priority setting techniques and able to factor in issues such as equity.

7. A priority-driven, policy relevant research program	Not reactive but anticipates need. Is able to formulate research questions, develop a proposal to answer those questions and execute the study.

8. An investigator-led research program	Not reactive – sees gaps in the available knowledge and tools, is able to develop a research plan to fill these gaps and secure funding to support the research agenda.

All survey participants indicated that economics and health economics have the potential to contribute concepts and techniques to address a wide range of issues faced by health system decision-makers. All but one of the departments of health we surveyed reported currently making use of health economics expertise to inform decision-making. Mechanisms used to secure this advice varied among the departments of health, over time and with the type of task, with most organisations using a mixture of in-house and external sources (Table 2). The range of options seen in practice extended from sourcing services from external groups (*n *= 2) to well- structured, specialist units within the health department (*n *= 3). Most departments used more than one mechanism at any time. In general, however, the number of in-house positions for health economists (or economists) was few. Two departments had developed specialist training programs to train health economists–only one of which continues today. Both these programs offered a combination of a university qualification in health economics and a period of work-based learning.

**Table 2:** Description of organisational mechanisms, both internal and external to the organisation, used to meet health economics needs by departments of health in Australia, 2004.

**Organisational mechanism**	**Description**
**Internal**	

Position descriptions require qualifications that include an appreciation of economics or health economics in the coursework	Many position descriptions in health require a qualification that includes some introduction to economics or health economics eg Masters of Public Health or Masters of Health Administration.

Staff training (e.g., the NSW Public Health Officer Training Program)	Short courses in health economics of varying duration and intensity, with or without final assessment of participants or accreditation of the courses.

Generalist staff with economic qualifications	Staff have a degree in economics but are not specialist health economists nor does their position require this qualification.

Specialist health economics training programs	Structured training programs to develop specialist health economists.

Health economist positions	Position description requires a qualification in health economics.

Health economics units	A team of health economists (with or without other disciplines) of varying size with well developed roles and functions to support decision making.

**External**	

Consultancy for services	Services of a scale that do not require contracted arrangements, usually for specific tasks, where expertise was sought through professional and personal networks.

Contract research	Contracts whose size does not warrant a competitive tendering process sometimes met by a preferred provider.

Contract research by tender	Contracts developed and let by competitive tender, usually filled by private providers or the academic sector.

Collaborative research centres	University professorial chairs or research centres established with funding that secures the focus of the work in whole or part to meet health service needs.

Three of the four other government departments approached in NSW had internal economics units. One department had developed guidelines to support their staff to collaborate effectively with their economics unit, and these guidelines were accompanied by workshops and training sessions. These departments supplemented this internal capacity with external expertise.

Table 3 describes the potential links between the mechanisms and the functions they fulfil as seen by the authors. The first row presents the mechanisms, with the internal (in-house) arrangements to the left and the external (out-of-house) sources of support to the right. The left-hand column presents the eight health economic functions, arranged in increasing order of technical complexity from top to bottom. Shaded boxes denote the functions that are best met by each particular mechanism.

**Table 3:** Organisational mechanisms used by departments of health in Australia in 2004 to secure health economics advice and the economic functions that these mechanisms might serve.

	**Internal Mechanisms**						**External Mechanisms**			
**Organisational mechanism Function**	Qualification s include appreciation of economics or health economics in coursework.	Staff training/NSW Public Health Officer Training Program	Staff with economic qualifications	Specialist H/E training programs	Health economist position	Health economics units	Consultancy for service	Contracted advice	Contracted advice by tender	Collaborative research centres

1. Appreciation of how economics fits into multi- disciplinary analysis of public health problems	Y	Y	Y	Y	Y	Y				

2. Advanced appreciation of economics concepts and methods, able to frame issues, formulate questions and obtain advice		Y	Y	Y	Y	Y	Y			

3. Economic analysis of (simple) problems and issues, requiring literature searches, appraisal, synthesis and interpretation.		Y	Y	Y	Y	Y	Y	Y	Y	

4. A capacity to respond quickly to emerging and emergency issues				Y	Y	Y				

5. Conducting economics evaluations and other studies, with appropriate methods			Y	Y	Y	Y	Y	Y	Y	Y

6. Application of economic findings to priority setting, emerging issues and decision- making					Y	Y	Y	Y	Y	Y

7. A priority- driven, policy relevant research program					Y	Y		Y	Y	Y

8. An investigator-led research program										Y

## Discussion

Our survey revealed a wide range of organisational mechanisms that have been used to secure health economics and economics advice by Australian governments. It also linked these mechanisms with the type of health economic functions that governments require to inform decision-making for policy and planning purposes. Our findings suggest that while health economics is established as part of the specialist public health workforce, the role is perhaps not as well-integrated into public health as other specialist areas, such as biostatistics. As further evidence of this, health economics was again raised in the most recent review of PHERP as a specialty area that still requires development [22].

Depending upon the type of health economic task at hand, health agencies use a number of different arrangements to meet that need. However, as observed by survey participants, not all the mechanisms will meet a given need equally well. Some questions that are commonly raised by government–for example, the cost and likely effect of interventions being implemented locally–can be difficult for external groups to answer, as much of the information required is held within the organisation. Further, government seeks answers to very practical questions and this may not readily correspond to the interests or expertise of external groups. None of the external mechanisms and very few of the internal mechanisms identified offer health services a true 'surge capacity' of health economics skills. Surge capacity can be required to provide, for example, advice on options for outbreak control [26] or to respond to urgent requests for budgeting options. There would be merit in investigating how well each of the mechanisms addresses different needs and to introduce a grading of usefulness to the responses.

Health departments varied also in the ease with which they were able to employ each mechanism. For example, successfully sourcing health economics capacity through external mechanisms is dependent upon suitable providers being identified and available. One jurisdiction noted that there were few local external providers from whom they could purchase these services. Establishing contracting arrangements through tender also takes time, and often advice is required quickly. Consequently, to streamline the process of contracting research, two jurisdictions had used 'preferred providers'; that is, small panels of pre-identified experts who were available to provide advice in areas of specialist knowledge. Membership of these panels was established through tendering processes.

We see in the mechanisms that have been identified both *structures*, such as position descriptions and contracting with experienced economists located in external centres, and *strategies *such as training. Structures ensure that people with the right skills are in place and are accessible, while strategies such as professional development provide opportunity for the development of skills required by the organisation for future capacity.

Training is also being used to ensure that staff responsible for accessing health economics expertise are efficient commissioners and users of that expertise, whether in-house or external. This was illustrated by a non-health department in NSW that had developed guidelines and training. Creating 'informed consumers' of health economics information is also an objective of the health economics module in the NSW Public Health Officer Training Program. This requires trainees to demonstrate a level of competence in health economics. We see similar developments internationally. For example, the National Institute of Health and Clinical Excellence in the UK has developed guidelines and frameworks for use by non-economist staff to ensure that cost-effectiveness questions are approached in a systematic way [27]. Bringing health service staff in contact with health economists also potentially builds informal collegial networks. The importance of these kinds of networks to building links between policy and evidence were described by Nutbeam [1]. Personal networks were described in one jurisdiction as enabling some staff to access advice from outside the organisation on a pro bono basis.

The mixture of structure and strategy points to important complementarities that exist among the mechanisms identified. One example of this is the provision of training programs–which requires access to competent trainers–who may also be the providers of high-level external expertise and advice to health departments.

The investigators are aware of at least two mechanisms for obtaining advice that were not described by participants. These were both external mechanisms. The first is where a formal agreement ensures that a quantum of advice is available on demand, provided either by an expert or a group of experts working either pro-bono or for a nominal fee. This is the mechanism that the NHMRC employs with its working groups and advisory groups. The second mechanism is where an external economist is retained to provide an agreed number of days' service.

A recent systematic review of the use of health economics by health authorities in the UK has questioned the capacity of health economics to assist government and concluded that more needed to be done '... to ensure alignment between the objectives assumed in economic analyses and the objectives facing decision-makers in reality'[18]. This need highlights another gap revealed by Table 3, that few mechanisms allowed health economics to continue to develop as a field through methodological research. This function is most likely to be met through specialist research centres and unlikely to be supported through funding that is limited to answering specific service-based questions. Consequently, health departments need to be aware of the other objectives of external research groups lest the sustainability and utility of these groups and their capacity to meet more immediate, policy-relevant needs is diminished.

In conclusion, the potential offered by health economics was first recognised more than 30 years ago. Since then, the need for health managers to be informed by a wide range of skills including economics has been emphasised and re-emphasised [1,22]. Most recently, Prime Minister Rudd, in an address to his heads of department and senior bureaucrats, stated: 'Policy innovation and evidence-based policy-making is at the heart of being a reformist government.' To achieve this end one of the seven elements of his government's vision for the Australian public service is developing evidence-based policy-making processes [28]. It is encouraging therefore that departments of health across Australia are looking to engage with health economics and have a variety of means of doing so available to them. We have also demonstrated that it is possible to describe an organisation's need for specialist services as a set of functions or competencies and then identify the range of mechanisms through which those functions were met.

## Competing interests

The authors declare that they have no competing interests.

## Authors' contributions

The study was originally conceived by LM in consultation with LK and AS. Study design was agreed by all three authors. Interviews and initial analysis were completed by LK, with comments and contributions from LM and AS. First draft of the manuscript was completed by LM based on a report compiled by LK. All authors contributed to the re-writing of the manuscript.
